# Supplementary material for: Evaluation of opportunities to implement community-wide mass drug administration for interrupting transmission of soil-transmitted helminths infections in India
Source: PLoS Negl Trop Dis. 2023 Mar 10;17(3):e0011176. doi: 10.1371/journal.pntd.0011176 (PMC10004831; doi:10.1371/journal.pntd.0011176)
Supplement: S1 File — (DOCX) [file pntd.0011176.s001.docx]

**Supporting File 1**

**Annex 1 – *State Organizational Readiness Survey***

| Survey Item | Survey Response | | | | | |
| --- | --- | --- | --- | --- | --- | --- |
| 1. In my experience, India's national neglected tropical disease (NTD) policy supports implementation of community-wide mass drug administration (MDA). | Disagree | Somewhat disagree | Unsure | Somewhat agree | Agree | Not enough information to answer |
| 1. In my experience, the NTD program leadership at the National level is effectively implementing community-wide MDA programs in India. | Disagree | Somewhat disagree | Unsure | Somewhat agree | Agree | Not enough information to answer |
| 1. In my experience, the National NTD Implementation Plan is currently being implemented in my state as intended. | Disagree | Somewhat disagree | Unsure | Somewhat agree | Agree | Not enough information to answer |
| 1. In my experience, the NTD program leadership at the state-level is effectively implementing community-wide MDA programs. | Disagree | Somewhat disagree | Unsure | Somewhat agree | Agree | Not enough information to answer |
| 1. In my experience, the NTD program leadership at the district level is effectively implementing community-wide deworming programs in India. | Disagree | Somewhat disagree | Unsure | Somewhat agree | Agree | Not enough information to answer |
| 1. I have observed that India's National NTD Implementation Plan provides sufficient guidance for implementing community-wide MDA programs, such as lymphatic filariasis (LF). | Disagree | Somewhat disagree | Unsure | Somewhat agree | Agree | Not enough information to answer |
| 1. I have observed that there is a collaborative network of external stakeholders (e.g., NGOs or technical/ financial partners) that would support community-wide deworming for STH in my state. | Disagree | Somewhat disagree | Unsure | Somewhat agree | Agree | Not enough information to answer |
| 1. I believe that my state needs to eliminate the transmission of STH. | Disagree | Somewhat disagree | Unsure | Somewhat agree | Agree | Not enough information to answer |
| 1. I have observed that my co-workers generally believe that my state needs to eliminate the transmission of STH. | Disagree | Somewhat disagree | Unsure | Somewhat agree | Agree | Not enough information to answer |
| 1. My state has the key resources necessary to effectively implement community-wide deworming for STH. | Disagree | Somewhat disagree | Unsure | Somewhat agree | Agree | Not enough information to answer |
| 1. I believe that community-wide deworming can eliminate the transmission of STH in my state. | Disagree | Somewhat disagree | Unsure | Somewhat agree | Agree | Not enough information to answer |
| Please explain your answer for the above question (#10) | | | | | | |
| 1. I have observed that my co-workers generally believe that community-wide deworming can eliminate STH transmission in my state. | Disagree | Somewhat disagree | Unsure | Somewhat agree | Agree | Not enough information to answer |
| 1. I am supportive of implementing community-wide deworming for STH in my state. | Disagree | Somewhat disagree | Unsure | Somewhat agree | Agree | Not enough information to answer |
| 1. In my opinion, my co-workers will be supportive of implementing community-wide deworming for STH. | Disagree | Somewhat disagree | Unsure | Somewhat agree | Agree | Not enough information to answer |
| 1. In my experience, MOHFW leadership at the National level are generally receptive to new ideas or pilot projects. | Disagree | Somewhat disagree | Unsure | Somewhat agree | Agree | Not enough information to answer |
| 1. How often do your supervisors generally feel comfortable receiving feedback and recommendations from you or your colleagues on how to improve program implementation? | Never | Rarely | Occasionally | Often | Always | Not enough information to answer |
| 1. How often do you present new ideas to your supervisor? | Never | Rarely | Occasionally | Often | Always | Not enough information to answer |
| 1. It is challenging to present new ideas to my supervisor. | Never | Rarely | Occasionally | Often | Always | Not enough information to answer |
| 1. How often do your subordinates generally feel comfortable providing feedback and recommendations to you or your colleagues on how to improve program implementation? | Never | Rarely | Occasionally | Often | Always | Not enough information to answer |
| 1. Community-wide deworming for STH is not necessary for my state | Disagree | Somewhat disagree | Unsure | Somewhat agree | Agree | Not enough information to answer |
| 1. Community-wide deworming will not be able to stop transmission of worms in my state. | Disagree | Somewhat disagree | Unsure | Somewhat agree | Agree | Not enough information to answer |
| 1. I believe that MOHFW personnel within my state can deliver community-wide deworming with high coverage. | Disagree | Somewhat disagree | Unsure | Somewhat agree | Agree | Not enough information to answer |
| 1. Ministry of Education personnel that I work with on school or child interventions will likely support community-wide deworming for STH. | Disagree | Somewhat disagree | Unsure | Somewhat agree | Agree | Not enough information to answer |
| 1. I believe that my state can adapt the existing school deworming program (NDD) for community-wide deworming without too much difficulty. | Never | Rarely | Occasionally | Often | Always | Not enough information to answer |
| 1. The current implementation context within my state would negatively affect the ability of the state NTD program to deliver community-wide deworming for STH. | Never | Rarely | Occasionally | Often | Always | Not enough information to answer |
| 1. How often have you encountered difficulty in moving funds within the state-level for a community-based program? | Never | Rarely | Occasionally | Often | Always | Not enough information to answer |
| 1. How often have you observed difficulties with having enough funding from the National level to support implementation of community-based programs in your state? | Never | Rarely | Occasionally | Often | Always | Not enough information to answer |
| 1. How often do you encounter difficulties with having enough funding at the district level to implement community-based programs? | Never | Rarely | Occasionally | Often | Always | Not enough information to answer |
| 1. In my experience, drug distributors are given sufficient financial and/or non-financial incentives for administering community-wide MDA. | Disagree | Somewhat disagree | Unsure | Somewhat agree | Agree | Not enough information to answer |
| 1. I am not worried about whether my state has sufficient future funding for community-wide MDA programs. | Disagree | Somewhat disagree | Unsure | Somewhat agree | Agree | Not enough information to answer |
| 1. I have observed that deworming medicines are distributed from the state-level to local levels without too much difficulty. | Disagree | Somewhat disagree | Unsure | Somewhat agree | Agree | Not enough information to answer |
| 1. How often have you observed delays in the arrival of drugs for MDA programs due to supply chain problems? | Never | Rarely | Occasionally | Often | Always | Not enough information to answer |
| 1. My state currently has the resources and tools needed to develop high-quality sensitization and education materials for community-wide deworming for STH. | Disagree | Somewhat disagree | Unsure | Somewhat agree | Agree | Not enough information to answer |
| 1. My state will need additional training of NTD personnel to effectively deliver community-wide deworming for STH. | Disagree | Somewhat disagree | Unsure | Somewhat agree | Agree | Not enough information to answer |
| 1. Additional supervisors are needed within the state to coordinate the delivery of community-wide deworming for STH. | Disagree | Somewhat disagree | Unsure | Somewhat agree | Agree | Not enough information to answer |
| 1. There is low motivation amongst state NTD personnel to implement community-wide deworming for STH. | Disagree | Somewhat disagree | Unsure | Somewhat agree | Agree | Not enough information to answer |
| 1. I believe that NTD personnel in my state have the skills needed to implement a new community-wide deworming program. | Disagree | Somewhat disagree | Unsure | Somewhat agree | Agree | Not enough information to answer |
| 1. In my experience, there is an effective program in my state for training drug distributors on how to deliver community-wide deworming. | Disagree | Somewhat disagree | Unsure | Somewhat agree | Agree | Not enough information to answer |
| 1. In my experience, NTD personnel within the state have demonstrated that they can deliver other community-wide MDA programs (e.g., lymphatic filariasis, LF) with high coverage. | Disagree | Somewhat disagree | Unsure | Somewhat agree | Agree | Not enough information to answer |
| 1. How often are treatment data incorrectly recorded during implementation of community-wide MDA programs? | Never | Rarely | Occasionally | Often | Always | Not enough information to answer |
| 1. I know of at least one community health program that could be used to deliver community-wide deworming for STH. | Disagree | Somewhat disagree | Unsure | Somewhat agree | Agree | Not enough information to answer |
| If yes, please list the community health programmes that can be used to implement community wide deworming for STH. | | | | | | |
| 1. In my experience, local drug distributors have the skills to effectively implement community-wide deworming for STH. | Disagree | Somewhat disagree | Unsure | Somewhat agree | Agree | Not enough information to answer |
| 1. How often are community members in your state resistant to community-wide MDA programs? | Never | Rarely | Occasionally | Often | Always | Not enough information to answer |
| 1. If the National government changes the policy to implement community-wide deworming for STH, I believe that my state is ready to implement it for the first time. | Disagree | Somewhat disagree | Unsure | Somewhat agree | Agree | Not enough information to answer |

**Annex 2. State Qualitative Research In-Depth Interview Guide**

1. What advantages does community-wide deworming for soil-transmitted helminths (STH) have compared to school-age deworming programs? What disadvantages?
2. What kind of information or evidence is available that shows whether or not community-wide deworming for interrupting transmission of STH will work in your state? Is this evidence strong or weak?

*Follow-up questions:* *What evidence are available from your state?*

1. If you were to implement a community deworming program in your state, what adaptations to the existing NDD program would be needed to achieve high coverage of community members of all ages? Do you think you will be able to make these adaptations? Why or why not? What aspects of the NDD program should not be adapted?

*Follow-up question:* *Are there any current challenges related to the drug supply chain for NDD in your state that would need to be addressed before adaptation of NDD?*

1. How challenging would it be to deliver community-wide deworming for STH in your State? Why? What challenges might community members face in participating in community-wide deworming for STH?

*Follow-up question:* *What could be done to overcome these barriers?*

1. What are some key strategies to achieve high treatment coverage for community-wide deworming? How important do you think it is to verify coverage in an STH elimination campaign? How would a process for verifying coverage be incorporated into a routine community-wide deworming program?
2. What are the linkages between LF staff and NDD staff at different levels of the health system? How effective are these linkages?
3. What LF program activities could be leveraged for community-wide deworming for STH? Are there any other routine community-based programs that can be leveraged to deliver community-wide deworming for STH? How might integration of community-wide deworming affect these other existing programs?
4. How important do you think it is to implement community-wide deworming for STH compared to the other health priorities in your State?
5. What are other ministries or departments outside of Ministry of Health and Family Welfare that you think may need to coordinate in order to effectively implement a community-wide deworming program for STH? What role will they play? What kind of support will the State need from National government to effectively implement a community-wide deworming program for STH?

*Follow-up question*: *How supportive do you think government leadership, within any Ministry, will be if community-wide deworming for STH is implemented in your State?*

1. If you want to implement community-wide deworming for STH in your State, what changes in formal policies or guidelines will need to take place? Can you describe the process that will be needed to make these changes?
2. From your perspective, what are the unique costs of implementing community-wide deworming program for STH as compared to a school deworming program?
3. Do you think that community-wide deworming could interrupt the transmission of STH (i.e. stop the spread of intestinal worms) in your State? Why or why not?

**Annex 3. Example of program mapping data collection sheet**


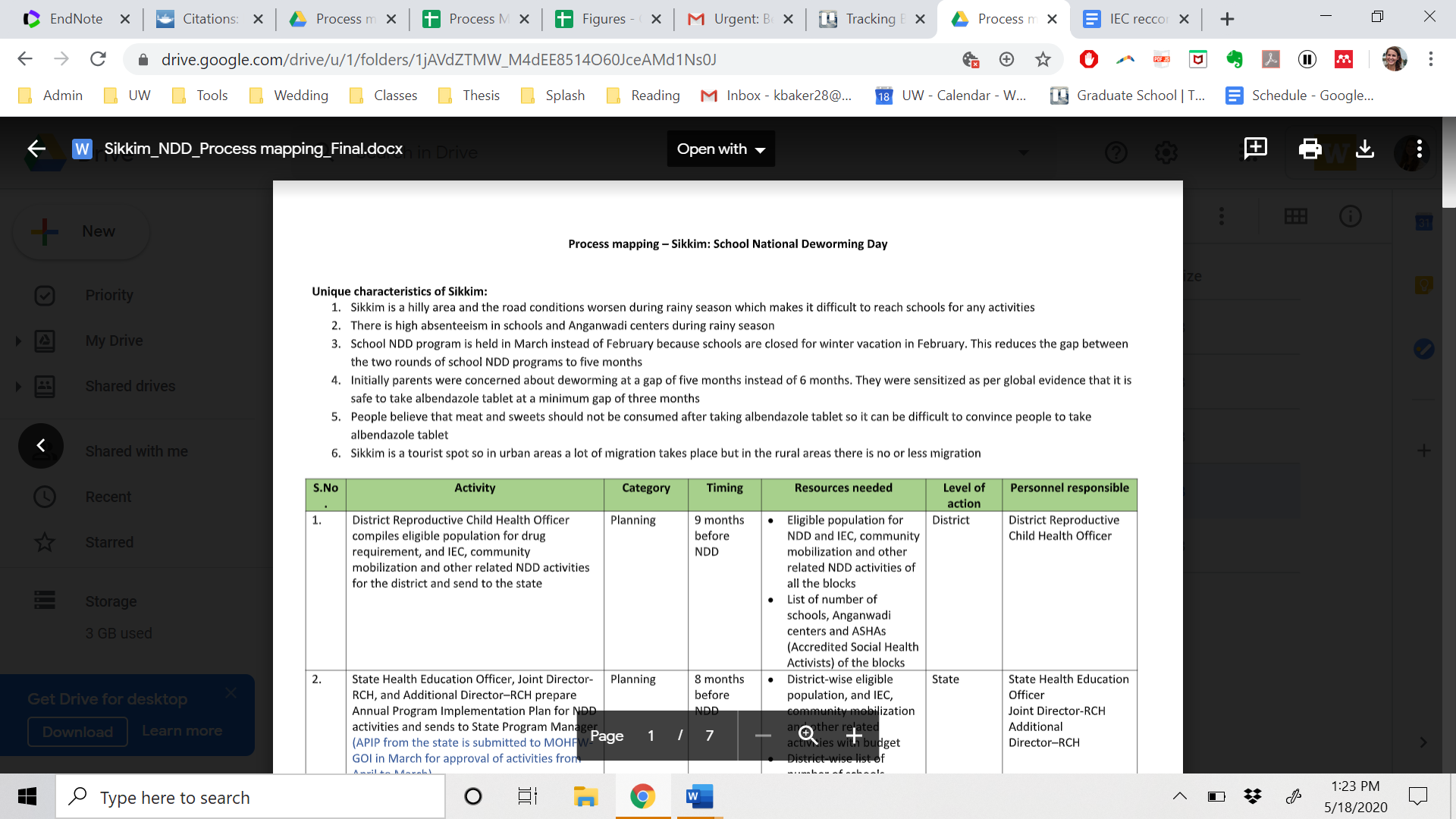


**Annex 4. Suggestions for key stakeholders to engage, made during qualitative interviews**

| Organizations | |
| --- | --- |
| Ministries | Ministry of Education |
|  | Ministry of Health & Family Welfare |
|  | Ministry of Human Resource Development |
|  | Ministry of Minority Affairs |
|  | Ministry of Rural Management & Development |
|  | Ministry of Social Justice & Empowerment |
|  | Ministry of Urban Development |
|  | Ministry of Water Resources/ Ministry of Drinking Water & Sanitation (Now merged under Ministry of Jal Shakti) |
|  | Ministry of Women & Child Development |
|  | Ministry of Youth Affairs and Sports |
|  | National Urban Health Mission (Under Ministry of Health & Family Welfare) |
| State-level or District-level | Central Public Works Department (Under Ministry of Urban Dev.) |
|  | Comprehensive Annual and Total Check-up for Healthy Sikkim (CATCH) |
|  | Department of Empowerment of Persons with Disabilities (Under Ministry of Social Justice) |
|  | Department of Water Resources (Under Ministry of Water Res.) |
|  | Community health workers (ASHAs, Anganwadi worker, Multipurpose worker) |
|  | Home-based newborn care programme (conducted by health workers) |
|  | Mahila Arogya Samiti |
|  | Public Health Engineering Department |
|  | Reproductive Child Health Programme |
|  | Scheduled Castes & Scheduled Tribe Department |
|  | School & Mass Education Department (Odisha) |
|  | Senior citizens programmes |
|  | Universities and Medical Colleges |
|  | Urban Nutrition Committee |
|  | Zila Parishad (District council) |
| Village-level | Community-based organizations |
|  | Municipal bodies |
|  | Panchayati Raj Institutes |
|  | School Teachers |
|  | Village Health & Sanitation Committee (Gaon Kalyan Samiti) |
| Other | Medical doctors (private & public) |
|  | Non-governmental organizations |
|  | Private sector/ corporations |
|  | Religious institutions |

**Annex 5. Community sensitization recommendations brainstormed by stakeholders during key informant interviews**

| IEC recommendations | |
| --- | --- |
| Important persons for sensitization | Leaders: Panchayat, religious, community |
|  | Active community members, self-help groups, other community groups |
|  | Teachers and children in school |
| Important persons for endorsement | Celebrities, collaboration with Department of Sports to have key messages communicated by famous athletes |
|  | Private medical doctors, Chief Medical Officers |
|  | Public officials, public figures |
|  | Media |
| Key messages to communicate | What the medication is, the health benefits of taking medication, disadvantages associated with not taking it, impacts on self, family, and greater society |
| Information Education Communication (IEC) methods | IPC (Interpersonal Communication) via one-on-one counseling, household counseling, focus-group discussions |
|  | Promotion at events, such as Village Health and Nutrition Day |
|  | Posting flyers/ health messaging on Village bulletin boards |
|  | National Deworming Day phone messages |
|  | IEC decorated vans |
|  | Folk theater, songs, cartoons |
|  | State-specific IEC videos |
|  | Mass media (TV, radio, community radio channels, newspapers, social media) |
| Equity & accessibility | Content that can be communicated to persons at all levels of literacy |
|  | Communicating content in multiple languages and preferably the population’s primary language |
|  | Partnering with Social Security of Empowerment of Person with Disabilities to make sure messaging is accessible (Odisha) |
