# Supplementary material for: Evaluation of opportunities to implement community-wide mass drug administration for interrupting transmission of soil-transmitted helminths infections in India
Source: PLoS Negl Trop Dis. 2023 Mar 10;17(3):e0011176. doi: 10.1371/journal.pntd.0011176 (PMC10004831; doi:10.1371/journal.pntd.0011176)
Supplement: Attachment — Submitted filename: Response to reviewers comments.docx [file pntd.0011176.s002.docx]

December 20, 2022

Maria Angeles Gómez-Morales and Eva Clark

Editors

PLOS Neglected Tropical Diseases (NTDs)

Re: PLOS Neglected Tropical Diseases submission (PNTD-D-22-01231)

Dear PLOS NTDs Editors,

We would like to sincerely thank you for reviewing and providing comments regarding our article entitled: "Evaluation of opportunities to implement community-wide mass drug administration for elimination of soil transmitted helminths in India." We have reviewed the editorial and reviewer comments and modified the manuscript in response to the reviewer feedback. Below, please find the comments and our point-by-point responses (in bold).

METHODS

-Are the objectives of the study clearly articulated with a clear testable hypothesis stated?
-Is the study design appropriate to address the stated objectives?
-Is the population clearly described and appropriate for the hypothesis being tested?
-Is the sample size sufficient to ensure adequate power to address the hypothesis being tested?
-Were correct statistical analysis used to support conclusions?
-Are there concerns about ethical or regulatory requirements being met?

**Reviewer #3:**

- The authors should define "elimination". WHO aim is to eliminate Soil-transmitted helminths as a health problem, what is not synonym as eliminating all infections.

**We have clarified across the manuscript that interruption of transmission is distinct from elimination as a public health problem.**

- I suggest the inclusion of a figure contabilizing and displaying the number of participants as well as the category they belong. The n of participants in Organizational Readiness Survey and in Qualitative Interviews should be displayed.
  **We have added the number of participants from each state for all the data collection streams (Table 1). Thank you for the suggestion.**

RESULTS

**Reviewer #3:**

- Table 1 should specify what the acronyms mean, maybe as a footnote. A flow diagram could be a complementary figure to this one.

**We have added the acronyms as suggested, thank you. This table includes differences between NDD and LF programs, and thus a simplified flow diagram is likely not possible, however we appreciate the suggestion.**

CONCLUSIONS

**Reviewer #3:**

- The authors should further discuss the following: How representative are these results? What is the n of the participants and their role? Do they have conflict of interest for cMDA? Did any biased could have occurred?

**A range of stakeholders at the state Ministry of Health & Family Welfare from officers directly responsible to training, executing, and monitoring the program to decision makers such as Director-Health Services were included in an effort to ensure representativeness of the sample (line # 41). None of the individuals reported a conflict of interest and no conflicts of interest were readily apparent. The state governments have been implementing the school-based deworming program with the aim of reducing anemia and other morbidities associated with STH. They were supportive of STH cMDA because of their aim to also reduce anemia among women in the reproductive age but expected some challenges in implementation.**

The authors should discuss the disadvantage of implementing cMDA, such as treatment resistance, already observed in few regions around the globe.
**In this manuscript we discussed the advantages and disadvantages of STH cMDA as reported by the study participants and have not discussed potential advantages or disadvantages that were not mentioned by the participants.**

EDITORIAL AND DATA PRESENTATION MODIFICATIONS

**Reviewer #3:**

- Table 1 should specify what the acronyms mean, maybe as a footnote. A flow diagram could be a complementary figure to this one.

**We have added the acronyms as suggested, thank you. This table includes differences between NDD and LF programs, and thus a simplified flow diagram is likely not possible, however we appreciate the suggestion.**

*GENERAL COMMENTS*

**Reviewer #1:**

- I think the paper is interesting but need a comprehensive rewriting especially on the introduction and in the discussion as many of the statements made by the authors are not supported by evidence.

**We have revised the introduction and discussion sections (detailed below) and also clarified the key findings accordingly.**

- For example: Line 33 he authors mention “evidence that may be possible to interrupt STH transmission” without bringing any support to this statement in any part of the paper; in reality there is no evidence that cMDA would interrupt transmission in areas where morbidity is relevant (and there are serious doubts if interrupting STH transmission in areas where there is no STH morbidity has any public health value).

**We have clarified that evidence for potential transmission interruption of STH with cMDA is based on mathematical models and have provided the references (line # 81).**

- Line 78 the author hypnotize  that the same intervention that has been successful for lymphatic filariasis would be effective STH also this statement is not supported by any evidence, firstly it is not possible compare the transmission of LF (that is inefficiently transmitted by mosquito) with a very efficient STH transmission through environmental contamination; secondly not a single country (or area of a country) where STH morbidity was significant  was ever able to interrupt transmission without an increase of the sanitation level that impede environmental contamination with human excreta.

**Yes, we agree that we cannot assume that a STH cMDA program would be as successful as the LF program. The point to be conveyed was that utilization of the demonstrably successful LF MDA delivery platforms for implementing STH cMDA could help achieve high treatment coverage (line# 96).**

- Line 442 cites WHO road map as source for “increasing interested of using expanded MDA to interrupt STH “this statement is not present in the WHO road map and therefore the statement is misleading

**We have corrected the statement (line# 468).**

- In conclusion I would suggest the authors to keep the focus on reporting the results of their investigation on the theoretical interest of local stakeholder in expanding the present target of deworming (the results are clearly presented) but avoiding to extrapolate on fantasies regarding the elimination of transmission that at the moment is not proved to be possible by drug alone in area of high endemicity.

**We have revised the statements in the manuscript to include stakeholders’ interest towards implementation of cMDA for interruption of STH transmission rather than STH elimination (line# 559).**

- In addition, in my opinion in the "limitations of the study" two important issues should be mentioned:
  - 1- the discussion about the financial coverage of the additional cost costs linked to cMDA has been conducted as additional resources could be made available from an higher level in MoH or donors. It would have been interesting to know if the stakeholder investigated would be ready to use part of their present budget to cover these additional cost (a positive attitude on this aspect would have been a more clear sign of feasibility).

**Thank you for the suggestion as this is a key finding. The stakeholders from all the three states indicated that their states may not have enough funding to implement cMDA indicating need for additional support from MoH. We have clarified this statement in the discussion (line# 481).**

- 2- The respondents were not informed about the additional risk linked to the expansion of the target group of stimulating drug resistance by increasing the coverage in individual that are at less risk of morbidity. The rick that their answers were not sufficiently informed should be mentioned.

**We asked participants about perceived challenges or negative aspects of expanding cMDA and have reported their responses accordingly but did not prompt them on either positive or negative potential outcomes so as not to bias their responses.**

**Reviewer #2:**

- This study objective is to determine the readiness of government organizations in three Indian states for transitioning from school-based MDA to cMDA. The prinicipal findings of the study indicate that the necessary conditions to implement this intervention are favorable in these three states. The methodology was thorough and the results are clearly explained. This type of studies offer insights on government's readiness to apply different interventions in order to aim for STH elimination and not only STH control.

**We thank the reviewer for their kind feedback.**

**Reviewer #3:**

- This study is very relevant for the field. The manuscript is very well written and easy to follow. However, the representativeness of this results in this region should be further explained and discussed.

**We have revised the discussion to include this suggestion (line # 541). The results may be generalizable to other Indian states considering transitioning from school-based deworming to cMDA. The process itself, of attempting to preemptively understand readiness and implementation processes, may also be replicable across Indian settings who are considering a transition from NDD to cMDA and seeking to identify context-specific opportunities and challenges to doing so.**

*FIGURE FILES*

- While revising your submission, please upload your figure files to the Preflight Analysis and Conversion Engine (PACE) digital diagnostic tool, <https://pacev2.apexcovantage.com>. PACE helps ensure that figures meet PLOS requirements. To use PACE, you must first register as a user. Then, login and navigate to the UPLOAD tab, where you will find detailed instructions on how to use the tool. If you encounter any issues or have any questions when using PACE, please email us at [figures@plos.org](mailto:figures@plos.org).

*DATA REQUIREMENTS*

- Please note that, as a condition of publication, PLOS' data policy requires that you make available all data used to draw the conclusions outlined in your manuscript. Data must be deposited in an appropriate repository, included within the body of the manuscript, or uploaded as supporting information. This includes all numerical values that were used to generate graphs, histograms etc.. For an example see here: <http://www.plosbiology.org/article/info%3Adoi%2F10.1371%2Fjournal.pbio.1001908#s5>.

**Anonymized data will be made available upon reasonable request, given that the participants involved in this study may be identifiable to individuals who have familiarity with NTD program personnel in India.**
