# Supplementary material for: Evaluation of opportunities to implement community-wide mass drug administration for interrupting transmission of soil-transmitted helminths infections in India
Source: PLoS Negl Trop Dis. 2023 Mar 10;17(3):e0011176. doi: 10.1371/journal.pntd.0011176 (PMC10004831; doi:10.1371/journal.pntd.0011176)
Supplement: Attachment — Submitted filename: Respense to reviewers .docx [file pntd.0011176.s003.docx]

February 13, 2023

Maria Angeles Gómez-Morales and Eva Clark

Editors

PLOS Neglected Tropical Diseases (NTDs)

Re: PLOS Neglected Tropical Diseases submission (PNTD-D-22-01231)

Dear PLOS NTDs Editors,

We sincerely thank the reviewers for the comments on our manuscript titled: "Evaluation of opportunities to implement community-wide mass drug administration for elimination of soil transmitted helminths in India." We have modified the manuscript in response to the reviewers’ feedback. Our point-by-point responses are given below.

Sincerely,

Sitara Swarna Rao Ajjampur

The Wellcome Trust Research Laboratory, Division of Gastrointestinal Sciences, Christian Medical College, Vellore, India

Phone: +91-416-228-2052

Fax: +91-416-228-2486

Email: [sitararao@cmcvellore.ac.in](mailto:sitararao@cmcvellore.ac.in)

RESPONSE TO REVIEWERS

Reviewer #1: see general comments

Reviewer #2:

The study's objective is clearly indicated in the abstract but not so clearly in the text. I suggest adding a sentence at the end of the introduction that explains the main objective.

- **Thank you for the suggestions. We have clarified the objective in the introduction (page 6: lines 97-102 as, *“In 2018, we carried out a multi-method study with government stakeholders in three Indian states, Goa, Sikkim and Odisha, to assess readiness for transitioning from school-based MDA to cMDA and identify opportunities to leverage existing infrastructure from other NTD programs like LF.”***

I believe the study design is appropriate for determining these regions' readiness to implement cMDA. It is difficult for a foreign reader to determine whether the regions chosen for study development have the highest STH prevalence. How would the results of this study differ if it was conducted in more remote areas? More information about the region's status should be included.

- **We chose states that had the highest probability of interrupting STH which was based six categories: STH epidemiologic profiles, helminth associated morbidity, presence of helminth transmission risk factors, community wide public health coverage indicators, and human resource capacity to engage in intensified STH programming (given on page 7: line # 119-133). For the better representation of the states, India was divided to four regions as - north, south, east/northeast, and west/central, and one state from the three regions were selected except the southern region as a STH community-wide MDA trial was already going on in the southern region in the state of Tamil Nadu. Therefore, we had one state from the other three regions in this study. The results of this study could differ across states conducting school-based deworming but also within state at the district level. On page 35: line # 546-549, we have included – *“However, the results of this study could differ across states implementing school-based deworming, and there could be variations in implementing cMDA at district-level within a state depending on the district-level STH prevalence and readiness to implement cMDA*.**

Reviewer #3: The comments and suggestions have been addressed.
**Thank you very much.**

RESULTS

Reviewer #1: see general comments

Reviewer #2: The study sites' institutional readiness is clearly described throughout the results. I find the results to be of adequate quality and clarity.

**We thank the reviewer for their kind feedback.**

Reviewer #3: The comments have been addressed.
**Thank you very much.**

CONCLUSIONS

Reviewer #1: see general comments

Reviewer #2: The data presented supports the authors' conclusions, and the findings are of great interest in order to provide knowledge of how effective a transition from school-based deworming to cMDA could be in an endemic region with prior cMDA infrastructure.

**Thank you very much.**

Reviewer #3: Regarding anthelmintic resistance, I would still suggest the authors to mention it during the discussion. This is probably the main and more relevant drawback of cMDA. Moreover, it has been studied in different regions of the world. Thus, should be at least mentioned.

**Thank you. In the discussion (page 33: lines # 513-517), we have included a statement in the discussion about drug resistance to STH in humans with reference as, *“The participants, however, did not discuss potential emergence of drug resistance as being a challenge to implementing cMDA for interrupting STH transmission. While well described in veterinary medicine, the clinical relevance of drug resistance markers to benzimidazoles detected in human STH infections is unclear, but remains a potential risk of cMDA.” (*Adegnika et.al, 2015 and Pilotte et al., 2022)**

EDITORIAL AND DATA PRESENTATION MODIFICATIONS

Reviewer #1: (No Response)

Reviewer #2: (No Response)

Reviewer #3: The comments have been addressed.
**Thank you very much.**

SUMMARY AND GENERAL COMMENTS

Reviewer #1: The review made by the authors is in my opinion acceptable except for two points in the introduction that are essential to put the results obtained in a context:

Line  58-61 The authors do not mention that the main reason why cMDA is not recommended by WHO is to avoid the development of drug resistance against the only presently available drugs. Parasite residence against albendazole and mebendazole is well documented in veterinary medicine where the entire population of infected animal has been treated for several years (1)

**Thank you for your suggestion. While rapid emergence of drug resistance in veterinary medicine has been well documented, there is no definitive evidence of clinically relevant benzimidazole resistance in human STH infections. While single nucleotide polymorphisms have been documented in *Necator*, *Trichuris* (where the efficacy of benzimidazoles is intrinsically lower) and *Ascaris* in multiple countries, the relevance remains unclear.**

**On page 33: lines # 513-516, we have included a statement in the discussion about drug resistance to STH in humans with reference as, *“The participants, however, did not discuss potential emergence of drug resistance as being a challenge to implementing cMDA for interrupting STH transmission. While well described in veterinary medicine, the clinical relevance of drug resistance markers to benzimidazoles detected in human STH infections is unclear but remains a potential risk of cMDA.” (*Adegnika et.al, 2015 and Pilotte et al., 2022)**

Line 78 80  The authors, mention that cMDA was successful in interrupting LF transmission without mentioning that the  LF transmission cycle is relatively inefficient (2) (microfilariae are transported by mosquito) while STH transmission is extremely efficient (3) (contamination of environment with parasite eggs)

**Thank you for your suggestion and the references. As felt appropriate, we have included them in the discussion with the references on page 34: lines# 536-539 as, *“However, the cycle of LF transmission is considered inefficient as few infectious microfilariae present in the mosquito penetrate a human host whereas risk of STH transmission is high because spread of contamination of soil with parasitic eggs.”***

REFERENCES  
1 Coles, G.C., et al  2006. The detection of anthelmintic resistance in nematodes of veterinary importance. Vet. Parasitol. 136, 167–185.
2- Streit T, Lafontant JG. Eliminating lymphatic filariasis: a view from the field. Ann N Y Acad Sci. 2008; 1136:53-63. 
3- Horiuchi S, Paller VG, Uga S. Soil contamination by parasite eggs in rural village in the Philippines. Trop Biomed. 2013; 30(3):495–503.

Reviewer #2: (No Response)

Reviewer #3: The comments have been addressed.


DO YOU WANT YOUR IDENTITY TO BE PUBLIC FOR THIS PEER REVIEW

Reviewer #1: No

Reviewer #2: No

Reviewer #3: No
